# The Crisis of Meaning in Aging and the Expansion of Medically Unjustified Euthanasia: A Psychiatric Imperative

**DOI:** 10.62641/aep.v53i5.2003

**Published:** 2025-10-05

**Authors:** Carlos De las Cuevas

**Affiliations:** ^1^Department of Internal Medicine, Dermatology and Psychiatry, Universidad de La Laguna, 38200 San Cristóbal de La Laguna, Spain

In recent years, some Western societies have entered a troubling phase in the discourse surrounding euthanasia: the progressive detachment of the practice from medical or psychiatric justification. Nowhere is this trend more visible than in the Netherlands, where the legal framework for euthanasia—already among the most permissive globally—is being challenged by proposals to offer assisted death to individuals aged 75 and over, even in the absence of terminal illness, intractable physical pain, or psychiatric disorder.

At the center of these debates are individuals who do not suffer from clinical pathology, but who experience life as devoid of purpose or satisfaction. As one elderly man stated publicly, “*Life, by itself, has no meaning; one has to give it one*”. This reflection, while existential in tone, resonates deeply with psychiatrists. In fact, it closely mirrors the inner dialogue of many patients suffering from depressive disorders, where meaninglessness, emotional fatigue, and hopelessness often dominate the subjective experience of self and time.

From a psychiatric perspective, the convergence of existential despair and euthanasia requests raises profound ethical and clinical questions. Diagnostic challenges are to be expected: differentiating between a structured depressive episode and an existential crisis can be complex, particularly in older adults who may present with subthreshold symptoms. Yet, the implications of error are enormous. If demoralization becomes medicalized as an indication for death, we risk enshrining hopelessness as a legitimate therapeutic outcome.

Empirical data support the idea that such existential states are dynamic, not static. For example, Breitbart *et al*. [1] have shown that interventions focused on meaning reconstruction—such as meaning-centered psychotherapy (MCP)—significantly reduce despair and improve quality of life, even in patients facing terminal illness. In non-terminal populations, therapies aimed at enhancing purpose, social connectedness, and narrative integration remain central to psychiatric care [2].

What we are witnessing, however, is a cultural shift where the erosion of meaning is treated not as a clinical or social challenge, but as a personal failure for which death is a permissible—perhaps even compassionate—response. This shift cannot be understood without examining the broader sociocultural context. Contemporary Western societies, dominated by the ideologies of individualism, productivity, and autonomy, have become increasingly ill-equipped to accommodate dependency, aging, and existential vulnerability [3].

In this environment, the elderly often find themselves alienated from the values that once gave structure to life: generativity, community, and transcendence. The loss of these anchors has been exacerbated by the breakdown of intergenerational households, the medicalization of suffering, and the decline of spiritual or philosophical narratives capable of sustaining meaning in the face of decline [4, 5].

From the vantage point of psychiatry, our duty is not to serve merely as neutral gatekeepers for euthanasia access. We are tasked with affirming the intrinsic worth of the person beyond their current capacity to produce, enjoy, or function independently. We are called to facilitate meaning-making, to reconstruct narratives, and to treat suffering—not to end it through elimination of the sufferer.

There are, of course, clinical situations where euthanasia in psychiatric illness has been debated. The literature contains a growing number of case reports and ethical analyses concerning treatment-resistant depression or chronic suicidality [6, 7]. Yet even in such cases, the standards for evaluation are high, multidisciplinary, and controversial. What is currently proposed in the Netherlands—euthanasia for existential weariness in otherwise healthy individuals—bypasses these safeguards and presents a new moral and clinical terrain altogether.

One cannot ignore the symbolic consequences of these changes. If we accept medically unjustified euthanasia, we send a collective message: that the absence of subjective meaning is equivalent to the presence of intolerable suffering, and that society will not only validate this assessment, but assist in its final resolution. This shift is not ethically neutral; it is a profound redefinition of care and of the human condition.

Rather than expanding access to euthanasia for non-medical reasons, our societies should invest in the infrastructures of meaning: relational care, palliative psychiatry, narrative medicine, and policies that affirm the dignity of aging. Psychiatry, in particular, has a central role to play—not in the facilitation of existential death, but in the restoration of existential life.

If we fail in this mission, we risk becoming silent collaborators in a culture that no longer knows how to live with vulnerability—and no longer sees the value in trying.
